# Implementing the US air quality standard for PM_2.5_ worldwide can prevent millions of premature deaths per year

**DOI:** 10.1186/s12940-016-0170-8

**Published:** 2016-08-23

**Authors:** Despina Giannadaki, Jos Lelieveld, Andrea Pozzer

**Affiliations:** 1 The Cyprus Institute, P.O. Box 27456, 1645 Nicosia, Cyprus; 2 Max Planck Institute for Chemistry, Hahn-Meitnerweg 1, 55128 Mainz, Germany; 3 King Saud University, Riyadh, 11451 Saudi Arabia

**Keywords:** Air quality, Outdoor air pollution, Fine particulate matter, PM_2.5_ standards, Premature mortality

## Abstract

**Background:**

Air pollution by fine aerosol particles is among the leading causes of poor health and premature mortality worldwide. The growing awareness of this issue has led several countries to implement air pollution legislation. However, populations in large parts of the world are still exposed to high levels of ambient particulate pollution. The main aim of this work is to evaluate the potential impact of implementing current air quality standards for fine particulate matter (PM_2.5_) in the European Union (EU), United States (US) and other countries where PM_2.5_ levels are high.

**Methods:**

We use a high-resolution global atmospheric chemistry model combined with epidemiological concentration response functions to investigate premature mortality attributable to PM_2.5_ in adults ≥30 years and children <5 years. We perform sensitivity studies to estimate the reductions in mortality that could be achieved if the PM_2.5_ air quality standards of the EU and US and other national standards would be implemented worldwide.

**Results:**

We estimate the global premature mortality by PM_2.5_ at 3.15 million/year in 2010. China is the leading country with about 1.33 million, followed by India with 575 thousand and Pakistan with 105 thousand per year. For the 28 EU member states we estimate 173 thousand and for the United States 52 thousand premature deaths in 2010. Based on sensitivity analysis, applying worldwide the EU annual mean standard of 25 μg/m^3^ for PM_2.5_ could reduce global premature mortality due to PM_2.5_ exposure by 17 %; while within the EU the effect is negligible. With the 2012 revised US standard of 12 μg/m^3^ premature mortality by PM_2.5_ could drop by 46 % worldwide; 4 % in the US and 20 % in the EU, 69 % in China, 49 % in India and 36 % in Pakistan. These estimates take into consideration that about 22 % of the global PM_2.5_ related mortality cannot be avoided due to the contribution of natural PM_2.5_ sources, mainly airborne desert dust and PM_2.5_ from wild fires.

**Conclusions:**

Our results reflect the need to adopt stricter limits for annual mean PM_2.5_ levels globally, like the US standard of 12 μg/m^3^ or an even lower limit to substantially reduce premature mortality in most of the world.

**Electronic supplementary material:**

The online version of this article (doi:10.1186/s12940-016-0170-8) contains supplementary material, which is available to authorized users.

## Background

Outdoor air pollution by fine particles ranks among the top ten global health risk factors that can lead to premature mortality [1]. Most of these particles originate from combustion engines, power plants, industry, household energy use, agriculture, biomass burning and natural sources like desert dust.

Epidemiological cohort studies, mainly conducted in the United States and Europe, have shown that the long-term exposure to PM_2.5_ (particles with an aerodynamic diameter less than 2.5 μm) is associated with increased mortality from cardiovascular, respiratory diseases and lung cancer [1–7]. It has been estimated that 70–80 % of premature deaths attributable to outdoor air pollution are due to ischemic heart disease and strokes, 15–25 % to chronic obstructive pulmonary disease and acute lower respiratory infections and about 5–6 % to lung cancer [8–10]. Fine particulates can cause health impacts even at very low concentrations [11–14]. Previously, no concentration level has been defined below which health damage can be fully prevented while the Global Burden of Disease (GBD) applies a PM_2.5_ threshold of 7.3 ± 1.5 μg/m^3^ [1].

The World Health Organization (WHO) ambient air quality guidelines suggest an annual mean PM_2.5_ concentration limit of 10 μg/m^3^ and 25 μg/m^3^ for the 24-hourly mean [11]. Populations in large parts of the world, especially in East and Southeast Asia and the Middle East, are exposed to levels of fine particulate pollution that far exceed the WHO guidelines. WHO reported that in 2012 outdoor air pollution was responsible for the deaths of 3.7 million people [9]. WHO also emphasizes that indoor and outdoor air pollution combined are among the largest health risk worldwide, both being of similar magnitude. Air pollution is considered the number one environmental cause of premature death in the European Union (EU) [15]. Air pollution additionally impacts the quality of life by causing non-lethal chronic respiratory problems including asthma. It causes loss of working days and high healthcare costs, affects climate and perhaps weather, harms ecosystems, limits visibility and damages monuments and buildings. The direct costs to the European Union society from air pollution, including damage to crops and buildings, are estimated at about €23 billion per year [15, 16].

In the United States (US), substantial reductions of particulate pollution have been achieved in the recent past. The Environmental Protection Agency (EPA) in December 2012 took further steps to reduce particle pollution by tightening the annual National Ambient Air Quality Standard for fine particles (PM_2.5_) from 15 to 12 μg/m^3^. Benefits of the US clean air act for 1970–1990 were estimated at a central value of $22.2 trillion compared to the implementation costs of $0.52 trillion [17, 18]. Many other countries have not yet enforced regulations to control PM_2.5_. Estimates of mortality and morbidity attributable to outdoor air pollution are useful to justify air quality control policies and help improve public health. The aim of this work is to evaluate the implementation of recent air quality standards for PM_2.5_ in the EU, US and other countries worldwide and to estimate the public health gains that could be expected if EU or US standards for long term exposure were adopted and enforced internationally. In Table 1 and section 4 we present information on the current regulations for annual mean PM_2.5_ concentrations that have been adopted in the EU, US and other countries. We also present proposed targets that have not been officially adopted, mainly in several Asian countries which contribute strongly to high PM_2.5_ levels and related mortality, and finally the World Health Organization Air Quality Guideline for annual mean PM_2.5_ levels.

**Table 1:** Summary of PM_2.5_ standards in selected countries (in μg/m^3^)

Countries/Unions	PM_2.5_ annual mean (μgm^-3^)	Status	Source
European Union	25	Adopted	EU, Air Quality Directive, 2008/50/EC
United States	12	Adopted	EPA Regulatory Actions, 2014
Canada	10	Adopted	Canadian Ambient Air Quality Standards, 2014
Colombia	25	Adopted	Green, J. and Sánchez S., 2012
Chile	20	Adopted	Green, J. and Sánchez S., 2012
Equador	15	Adopted	Green, J. and Sánchez S., 2012
El Salvador	15	Adopted	Green, J. and Sánchez S., 2012
Mexico	15	Adopted	Green, J. and Sánchez S., 2012
Puerto Rico	15	Adopted	Green, J. and Sánchez S., 2012
Rep of Dominica	15	Adopted	Green, J. and Sánchez S., 2012
Argentina (Buenos Aires)	15	Adopted	Green, J. and Sánchez S., 2012
Bolivia (La Paz)	10	Adopted	Green, J. and Sánchez S., 2012
Australia	8	Adopted	Australian Gov., Dep. of the Environment and Heritage
China (Beijing)	35	Proposed	CAI-Asia, Particulate Matter Standards in Asia, 2010
India	40	Proposed	CAI-Asia, Particulate Matter Standards in Asia, 2010
Japan	15	Proposed	Environmental Quality Standards in Japan, 2014
Pakistan	15	Proposed	CAI-Asia, Particulate Matter Standards in Asia, 2010
Bangladesh	15	Proposed	CAI-Asia, Particulate Matter Standards in Asia, 2010
Saudi Arabia	15	Proposed	Kingdom of Saudi Arabia: National Env. Standard, 2014
WHO	10	Guideline	World Health Organization Air Quality Guidelines 2005

## Methods

### Estimation of PM_2.5_ related mortality

To estimate premature mortality attributable to PM_2.5_ we used the following health impact function

1 $$ \varDelta \mathsf{Mort}={\mathsf{y}}_{\mathsf{o}}\cdot \mathsf{A}\mathsf{F}\cdot \mathsf{Pop} $$

Where *y*_*o*_ is the baseline mortality rate [8, 19, 20] of the population (*Pop*) exposed to air pollution. We used mortality data from the World Health Organization [21] for ischemic heart disease (IHD), cerebrovascular disease (CEV), chronic obstructive pulmonary disease (COPD), and lung cancer (LC) for the population above 30 year (≥30 year), and for acute lower respiration infection (ALRI) for children below 5 years (<5 years). We focused on the above detailed health outcomes to be consistent with the Global Burden of Disease 2010 study [1].

The corresponding population data have been obtained from the Columbia University Center for International Earth Science Information Network [22], available at high resolution (about 5 × 5 km^2^).

*AF* is the fraction of the disease burden attributable to the risk factor (here PM_2.5_). The attributed fraction is defined as

2 $$ \mathit{\mathsf{A}}\mathit{\mathsf{F}}=\left(\mathit{\mathsf{R}}\mathit{\mathsf{R}}-\mathit{\mathsf{1}}\right)/\mathit{\mathsf{R}}\mathit{\mathsf{R}} $$

*RR* is the relative risk of certain health impacts of the population exposed to outdoor PM_2.5_ air pollution. To estimate the global burden of disease attributable to PM_2.5_ we follow the same methodology as Lelieveld et al. [8], and apply the integrated health risk function from Burnett et al. [23], also used by Lim et al. [1] for the GBD in 2010.

3 $$ \mathit{\mathsf{R}}\mathit{\mathsf{R}} = \mathsf{1}+\mathit{\mathsf{a}}\left\{\mathsf{1}-\mathit{\mathsf{exp}}\left[-\mathit{\mathsf{b}}{\left(\mathit{\mathsf{X}}-{\mathit{\mathsf{X}}}_{\mathit{\mathsf{o}}}\right)}^{\mathit{\mathsf{p}}}\right]\right\} $$

**Fig. 1:**
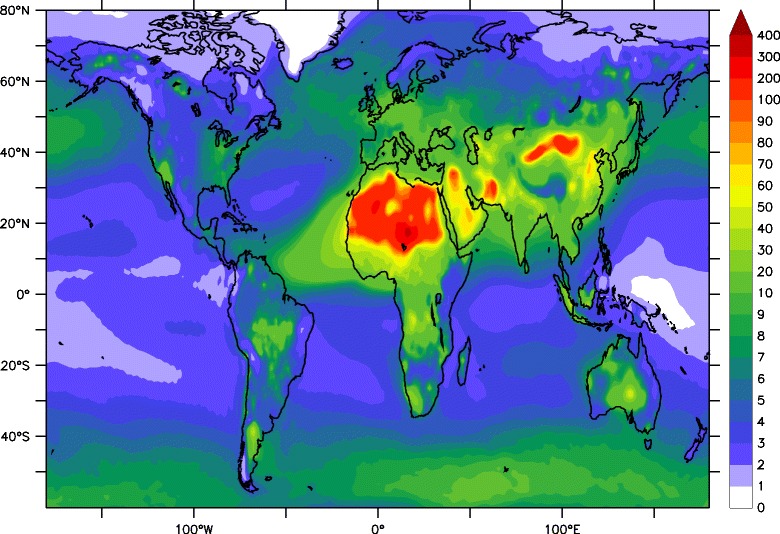
Model (EMAC) calculated PM_2.5_ concentrations (in μg/m^3^) in 2010

We refer to Burnett et al. [23] and Lelieveld et al. [8] for details on the exposure response models for the five disease categories. *X* is the annual mean PM_2.5_ concentration in 2010. We used the EMAC global atmospheric chemistry – general circulation model to simulate annual mean PM_2.5_ concentrations [24] (Fig. 1). EMAC comprises sub-models that represent tropospheric and lower stratospheric processes and their interaction with oceans, land and human influences [24–27]. We obtained results for the year 2010, applying monthly varying emissions from EDGAR - the Emission Database for Global Atmospheric Research [26]. We apply the same methodology as Lelieveld et al. [8] to estimate the premature mortality in 2010, combining all aerosol types that contribute to PM_2.5_, and using the same lower limits as Burnett et al. (around 7.3 μg/m^3^ depending on the disease category) for the background concentration *X*_*o*_ below which no impact is assumed [23]. To have a measure of the uncertainty range for the mortality estimations, we mainly use the lower and upper bound of RR to calculate the minimum and maximum AF and mortality.

Details about the EMAC atmospheric chemistry model, comparison of the output to in situ and remote sensing observations, and output robustness is available in Jöckel et al. [25], Lelieveld et al. [8, 28], Pozzer et al. [24, 26, 27] and references therein.

To assess the impact of applying air quality standards by the EU, US and other countries for PM_2.5_ pollution we performed sensitivity calculations where we set these standards as upper limit for the variable *X* in equation 3, thus assuming they are strictly implemented.

### PM_2.5_ standards and guidelines

European Union: The directive on ambient air quality and cleaner air for Europe [29] defines “objectives for ambient air quality designed to avoid, prevent or reduce harmful effects on human health and the environment as a whole”*.* Under this directive EU member states are required to reduce the exposure to PM_2.5_ in urban areas on average by 20 % in 2020 relative to 2010 levels. The states are obliged to bring exposure levels below 20 μg/m^3^ by 2015 in these areas. Throughout their territory member states will need to respect the annual mean PM_2.5_ limit value of 25 μg/m^3^. This value must have been achieved by 2015. In the air quality directive a PM_2.5_ reference level of 25 μg/m^3^ is set, initially as target value to be met by 2010 and as limit value to be met by 2015. In a second stage a lower limit of 20 μg/m^3^ must be met by 2020. Information from PM_2.5_ monitoring stations is still limited and needs to be extended to verify full implementation of the directive.

United States: In December 2012, the US Environmental Protection Agency (EPA) tightened the air quality standards for PM_2.5_ to improve air quality and public health. The primary annual mean PM_2.5_ concentration limit was lowered from 15 μg/m^3^ to 12 μg/m^3^. EPA has issued a number of regulations to meet the revised standard. EPA estimates that meeting the annual fine particle standard of 12 μg/m^3^ will provide health benefits at an economic value estimated at $4 to $9.1 billion per year in 2020, which translates into a return of $12 to $171 for every dollar invested in pollution reduction. Estimated annual costs of implementing the standard are $53 to $350 million [30].

Canada: On May 2013, the Canadian Environmental Protection Act established for the first time a long-term annual target for PM_2.5_ of 10 μg/m^3^ to be met by the year 2015, and a more stringent value of 8.8 μg/m^3^ to be met by 2020 [31].

Australia: On June 1998, the National Environment Protection Council (NEPC) in Australia set national standards for annual mean PM_2.5_ to not exceed 8 μg/m^3^, which is by far the strictest national limit worldwide. The standards should have been met by the year 2008 [32].

Other countries: We have conducted an internet search for information about regulations of PM_2.5_ in other countries with enhanced particulate pollution, and found that for many countries in Asia, Africa and Latin America records and data are scarce. In Latin America only few countries have set national ambient air quality standards. Colombia adopted a limit of 25 μg/m^3^ for annual mean PM_2.5_. Chile set a level of 20 μg/m^3^, while Ecuador, El Salvador, Mexico, Puerto Rico and the Dominican Republic have adopted a standard of 15 μg/m^3^. Provinces in Argentina and Bolivia implement regulations based on their own standards. Buenos Aires set a value of 15 μg/m^3^ annual mean PM_2.5_, and La Paz 10 μg/m^3^ [33].

The “Clean Air Initiative for Asia” [34] was established in 2001 as the premier air quality network for Asia by the Asian Development Bank, World Bank, and USAID. Its mission is to promote ways to improve air quality in Asian cities and provide information on air quality monitoring, status, and trends, and also on national air quality standards in Asian countries. While several Asian countries have adopted a standard for PM_10_, more is needed in the development of a PM_2.5_ standard. In China an upper annual mean PM_2.5_ limit of 35 μg/m^3^ is suggested for the Beijing municipality area and Hong Kong special administrative region (SAR). The reported annual mean PM_2.5_ concentration in Beijing is 89.5 μg/m^3^, far exceeding the national standard (https://www.chinadialogue.net/blog/6686-Beijing-passes-law-to-curb-air-pollution/en). Zheng et al. (2014) [35] analyzed long-term measurement data in Central Beijing, indicating an annual mean concentration of about 100 μg/m^3^. In India an upper annual mean PM_2.5_ limit of 40 μg/m^3^ has been proposed, which has not been formally adopted. Japan, Pakistan, Bangladesh and Saudi Arabia propose a limit of 15 μg/m^3^ [36–38]. For other countries with high PM_2.5_ pollution and associated mortality, like Russia, Ukraine, Indonesia, Viet Nam, Japan, Thailand, Egypt, Turkey, Iran, Iraq, Nigeria, Sudan and Myanmar we could not find specific regulations.

World Health Organization Air Quality Guidelines (WHO AQG): The WHO guideline for long-term PM_2.5_ exposure is an annual mean concentration of 10 μg/m^3^. With this AQG WHO offers guidance in reducing the health impacts of air pollution, but they are neither standards nor legally binding criteria. Epidemiological studies have not identified thresholds below which adverse health effects do not occur, thus the guideline value cannot fully protect humans from health impacts [11, 39].

## Results

**Fig. 2:**
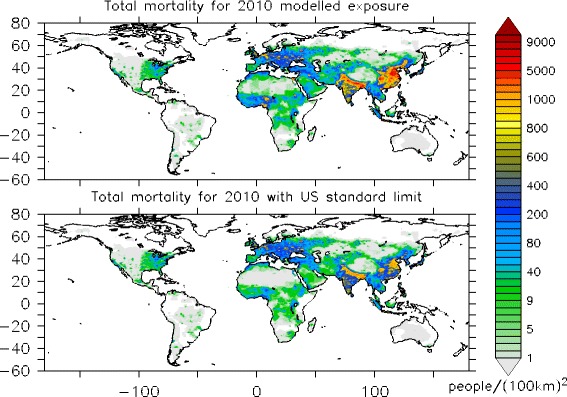
PM_2.5_ related premature mortality for the population <5 and ≥30 years old (in deaths/area of 100 × 100 km^2^). *Top*: year 2010. *Bottom*: Implementing the US standard of 12 μg/m^3^

We apply the exposure response model (Eq. 3) of Burnett et al. [23], to estimate the global and country level premature mortality due to CEV, IHC, COPD, and LC for the population ≥30 year, and due to ALRI for children <5 years in 2010, related to the long-term exposure to PM_2.5_. Consistent with Lelieveld et al. [8] for the year 2010 we estimate 3.15 million premature deaths (95 % confidence interval (CI95): 1.52–4.60 million) by PM_2.5_ worldwide, due to CEV (1.31 million), IHD (1.08 million), COPD (374 thousand), LC (161 thousand) and ALRI (230 thousand). Figure 2 (top) highlights the hot spot locations in red with high rates of premature mortality due to PM_2.5_ in 2010. The countries with the highest estimated premature mortality are China (1.33 million; CI95: 0.64–1.94 million), India (575 thousand; CI95: 277–840 thousand) and Pakistan (105 thousand; CI95: 51–153 thousand). For the EU our estimate is about 173 thousand (CI95: 83–253 thousand) with Germany ranking first (34 thousand), followed by Italy (19 thousand), France (17 thousand), United Kingdom (15 thousand), Romania (15 thousand) and Poland (14 thousand). Other countries in Europe with high premature mortality are Russia (67 thousand) and Ukraine (51 thousand). The United States ranks 7^th^ on the global list of premature mortality due to PM_2.5_ (Table 2) with about 52 thousand deaths in 2010 (CI95: 25–76 thousand). Table 2 shows the top 20 countries with highest PM_2.5_ related premature mortality in 2010, while Table 3 presents mortality data estimated for the 28 countries of the EU.

**Table 2:** Top 20 countries with highest annual premature mortality attributed to PM_2.5_ in 2010 for the population <5 and ≥30 years old and the corresponding mortality after the implementation of the EU and US air quality standards

Country	Year 2010 deaths (×10^3^)	EU limit (25 μgm^-3^) deaths (×10^3^)	US limit (12 μgm^-3^) deaths (×10^3^)
China	1327	910 (31)	416 (69)
India	575	502 (13)	294 (49)
Pakistan^a^	105	84 (20)	67 (36)
Nigeria^a^	89	78 (12)	76 (15)
Bangladesh	85	76 (11)	38 (55)
Russia	67	67 (0)	66 (1)
USA	52	52 (0)	49 (6)
Indonesia	51	48 (6)	33 (35)
Ukraine	51	51 (0)	49 (4)
Viet Nam	43	36 (16)	18 (58)
Germany	34	34 (0)	26 (24)
Egypt^a^	34	33 (3)	33 (3)
Turkey	31	31 (0)	25 (19)
Iran^a^	25	24 (4)	22 (12)
Sudan^a^	24	24 (0)	24 (0)
Japan	24	24 (0)	21 (13)
Myanmar	21	21 (0)	14 (33)
Italy	19	19 (0)	15 (21)
Iraq^a^	19	19 (0)	19 (0)
Thailand	18	18 (0)	15 (17)
World	3155	2600 (17)	1712 (46)

In parenthesis the % reduction in premature mortality

^a^In these countries PM_2.5_ is dominated by airborne desert dust

**Table 3:** Annual premature mortality attributed to PM_2.5_ in 2010 for the population <5 and ≥30 years old in the EU member countries and the corresponding mortality after the implementation of the EU and US air quality standards

Country	Year 2010 deaths (×10^3^)	EU limit (25 μgm^-3^) deaths (×10^3^)	US limit (12 μgm^-3^) deaths (×10^3^)
Germany	34	34	26 (24)
Italy	19	19	15 (21)
France	17	17	15 (12)
United Kingdom	15	15	14 (7)
Romania	15	15	12 (20)
Poland	14	14	10 (29)
Hungary	7.1	7.1	5.4 (24)
Spain	6.5	6.5	6.4 (2)
Czech Republic	6.5	6.5	4.3 (34)
Netherlands	4.7	4.7	2.9 (38)
Bulgaria	4.7	4.7	3.4 (28)
Belgium	4.4	4.4	2.9 (34)
Greece	3.9	3.9	3.1 (21)
Slovakia	3.7	3.7	2.7 (27)
Austria	3.0	3.0	2.4 (20)
Croatia	2.2	2.2	1.8 (18)
Lithuania	2.1	2.1	2.1 (0)
Portugal	1.8	1.8	1.8 (0)
Denmark	1.6	1.6	1.5 (6)
Latvia	1.3	1.3	1.3 (0)
Sweden	0.928	0.928	0.897 (3)
Slovenia	0.685	0.685	0.517 (25)
Ireland	0.538	0.538	0.538 (0)
Estonia	0.498	0.498	0.498 (0)
Finland	0.445	0.445	0.445 (0)
Malta	0.164	0.164	0.118 (28)
Cyprus	0.142	0.142	0.132 (7)
Luxemburg	0.106	0.106	0.078 (26)
EU total	173	173	138 (20)

In parenthesis the % reduction in premature mortality

Our global estimate of premature mortality due to long term exposure to PM_2.5_ (3.15M/year) agrees closely with the 3.22M/year estimate reported by the GBD study in 2010 [1] and the 3.24M/year estimate of Apte et al. [40]. Lelieveld et al. [28] estimated 2.2M/year for the global PM_2.5_ related mortality for 2005, which is 30 % less than our current estimate. This difference can be explained mainly by the new integrated health risk function and concentration response factors that we apply here and in particular also that we account for both anthropogenic and natural sources for PM_2.5_ in 2010, while Lelieveld et al. [28] accounted only for anthropogenic pollution in 2005. In addition, trends in PM_2.5_ concentrations and populations caused a significant increase in air pollution related deaths in densely populated countries like China and India. Further, in previous work premature mortality due to respiratory disease was attributed to O_3_ pollution, whereas more recently this has been subdivided into COPD by O_3_ and PM_2.5_. Hence the relative role of PM_2.5_ has increased at the expense of O_3_ in recent concentration exposure models.

In this work we also assess the contribution of natural sources of PM_2.5_, like desert dust, biomass burning (i.e., wild fires) and sea salt to premature mortality. Our estimates indicate that natural sources cause about 692 thousand deaths in 2010 (22 % of the total global mortality attributed to PM_2.5_). For the above estimations we assume that all PM_2.5_ particles with different composition, coming from different emission sources, are equally toxic. Based on a sensitivity study by Lelieveld et al. [8], who assumed that carbonaceous compounds are five times more toxic than inorganic and crustal compounds (e.g., dust) but maintaining the overall toxicity of total PM_2.5_, the contribution of natural sources to total mortality significantly reduces to about 460 thousand deaths in 2010 (15 % of the total premature mortality). Table 4 shows the contribution of PM_2.5_ from natural sources to the annual mortality for the countries that are mostly affected. In an earlier study we estimated premature mortality from cardiopulmonary diseases due to the long-term exposure to desert dust to be about 402T/year in 2005 [19]. For this estimate we used a linear health response function, and instead of the annual mean dust concentration we applied median values due to the episodic nature of desert dust outbreaks. In the same study we estimated 622 thousand deaths when we account for annual mean dust concentration.

**Table 4:** Top 20 countries with highest fraction of annual premature mortality attributed to natural sources of PM_2.5_ over total PM_2.5_ related mortality in 2010 for the population <5 and ≥30 years old

Country	PM_2.5_ deaths (×10^3^)	Natural sources deaths (×10^3^)	Fraction (%)
Sudan	24	24 (23)	100 (96)
Iraq	19	19 (18)	100 (95)
Saudi Arabia	14	14 (13)	100 (93)
Niger	13	13 (12)	100 (92)
Mali	9.4	9.3 (9.0)	99 (96)
Chad	7.4	7.3 (7.2)	99 (97)
Burkina Faso	9.3	9.1 (8.6)	98 (92)
Egypt	34	33 (31)	97 (91)
Cameroon	8.3	7.9 (7.2)	95 (87)
Ghana	9.3	8.7 (8.0)	93 (86)
D.R. Congo	15	13 (13)	87 (87)
Nigeria	89	76 (61)	85 (68)
Algeria	13	11 (11)	85 (85)
Morocco	13	11 (10)	85 (77)
Iran	25	21 (20)	84 (80)
Uzbekistan	11	7.8 (6.8)	71 (62)
Pakistan	105	65 (27)	62 (26)
India	575	94 (14)	16 (2)
Indonesia	51	8.2 (8.5)	16 (17)
China	1327	125 (46)	9 (3)
World	3155	692 (460)	22 (14)

In parentheses results of sensitivity calculations where carbonaceous aerosol compounds are assumed to be five times more toxic compared to inorganic and crustal compounds

### Sensitivity calculations

We present sensitivity calculations where we set different upper limits for the annual mean PM_2.5_ concentration (*X* in equation 1) based on air quality standards and regulations. To estimate potential reductions in mortality rates we take into consideration the deaths that cannot be avoided after implementation of the PM_2.5_ upper limits, due to the contribution of natural sources to the total PM_2.5_ and therefore to mortality (mainly airborne desert dust and natural biomass burning).

First, based on Table 1, we assume that all current national regulations and proposed limits for annual mean PM_2.5_ are fully implemented. The estimated global premature mortality is reduced by 9 % from 3.15 million to 2.86 million per year [CI95: 1.38-4.17M]. The main contributors to this reduction are the standards implemented in China causing about 16 % less deaths, Pakistan with 34 % less deaths, Bangladesh with 41 % less deaths and the US with 4 % less deaths.

In a second sensitivity calculation we apply the annual mean PM_2.5_ concentration of 25 μg/m^3^ as an upper limit, following the EU standard. We estimate 2.60 million [CI95: 1.25-3.80M] premature deaths per year globally; 17 % less compared to our base estimate for 2010 (Table 2). The estimated total and country level mortality within the EU remains almost unchanged, indicating that this standard is mostly met already. Our model results suggest that in many EU countries the annual mean total and anthropogenic PM_2.5_ concentrations are well below this limit (e.g., Scandinavia, Western Europe), thus the annual mean PM_2.5_ limit of 25 μg/m^3^ is too high to make a difference, and a reduction of mortality attributable to PM_2.5_ will require stricter limits. If the EU limit is applied in China, the main contributor to global PM_2.5_ related mortality, premature mortality could be reduced by 31 %, and about 417 thousand premature deaths would be avoided per year [CI95: 201-609T]. In India this limit could reduce premature mortality by about 13 % (73 T less deaths; CI95: 35-107T]. In a second stage the EU directive 2008/50/EC set a lower limit of 20 μg/m^3^ to be met by the year 2020. If we apply this limit in 2010 globally, mortality could be reduced by 26 % per year, still with a minor change within the EU. In China we estimate a reduction by 44 and 22 % in India (about 585 and 129 thousand less, respectively).

In a final sensitivity calculation we apply the limit of 12 μg/m^3^ based on the standard enacted in the US. According to our data, this limit could reduce the global premature mortality by 46 % compared to the 2010 estimates, from 3.15 [CI95: 1.52-4.60M] to 1.71 million deaths per year [CI95: 0.825-2.50M] (Table 2; Fig. 2, bottom), preventing about 1.44 million deaths/year. Our estimates indicate that in the United States the annual mortality could be reduced from 52 to 49 thousand per year [CI95: 24-72T], hence leading to a small improvement (by 4 %) in preventing mortality. If the EU would implement the 12 μg/m^3^ limit, instead of the 25 μg/m^3^, premature mortality could be reduced by 20 % to about 138 thousand per year [CI95: 66-201T], which is a considerable change; about 8.6 thousand deaths per year would be avoided in Germany, 4.1 thousand in Italy, 2.4 thousand in France, 1.2 thousand in the United Kingdom, 3.0 thousand in Romania, 4.3 thousand in Poland, 1.7 in Hungary, 2.2 in Czech Republic and 1.8 in Netherlands (Table 3). If the relatively strict US limit of 12 μg/m^-3^ would be applied in China, premature mortality could be reduced by 69 %, and about 911 thousand premature deaths would be avoided per year [CI95: 0.440-1.33M]. In India the implementation of the US upper limit concentration could reduce premature mortality by about 49 % and about 281 thousand deaths would be avoided per year [CI95: 136-411T]. In Pakistan and Bangladesh, he 3^rd^ and 5^th^ countries in the global ranking of 2010 PM_2.5_ associated mortality, the stricter US limit could reduce premature mortality by 36 % (about 38 thousand less deaths per year [CI95: 18-55T]) and 55 % (about 47 thousands less premature deaths per year [CI95: 23-69T]), respectively. Therefore, implementing the stricter US limit could make a significant difference (Table 2). In Nigeria, which is the 4^th^ ranking country in 2010 with an estimated 89 thousand deaths per year, PM_2.5_ is overwhelmed by natural sources mainly from Saharan desert dust, which contributes about 85 % to the total PM_2.5_ related mortality causing about 76 thousand deaths. The implementation of the US limit could hence only reduce mortality by 15 % (about 12 thousand less deaths per year [CI95: 6.1-18T]). Similarly, natural sources contribute strongly to PM_2.5_ and therefore to mortality in other countries mainly around the dust belt, an area that extends from North Africa across the Middle East and South Asia to East Asia (Table 4). For these countries it is not possible to meet the strict US limit, not even the EU limit, as high desert dust concentrations are dominant in large areas where the annual mean concentrations typically range from 20 μg/m^3^ to 200 μg/m^3^.

**Fig. 3:**
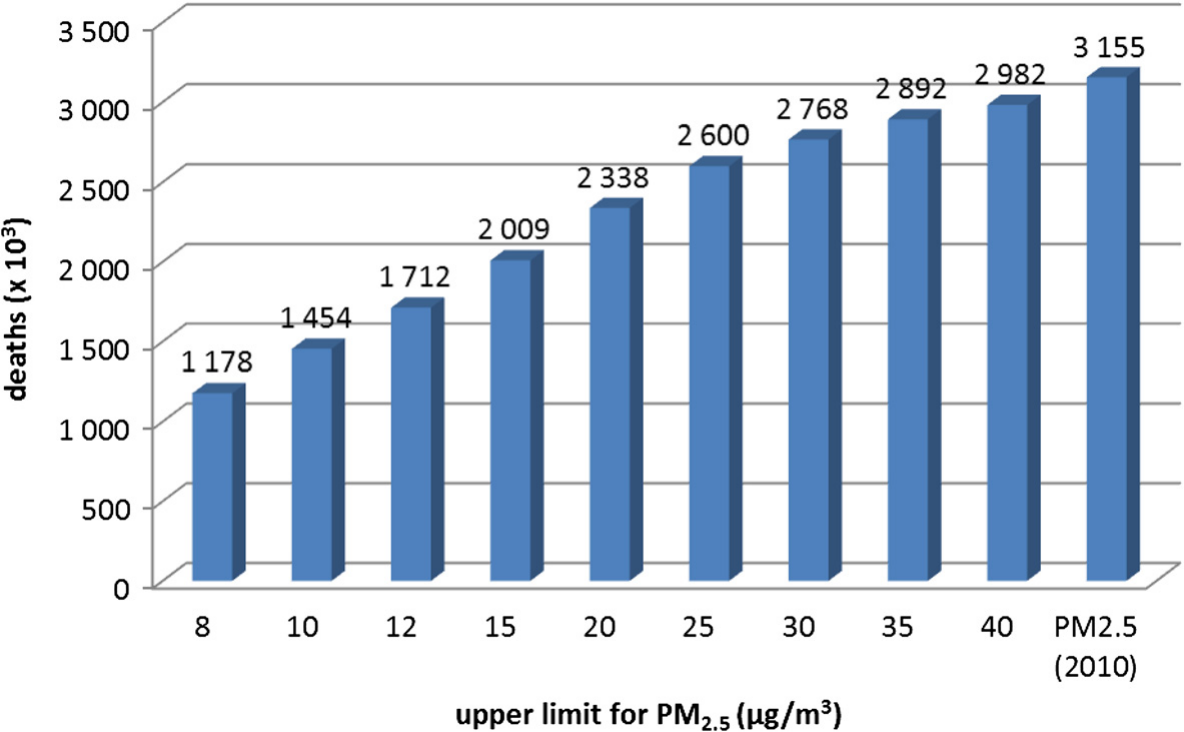
Global premature mortality attributed to PM_2.5_ for the population <5 and ≥30 years old, where different upper limits for annual mean PM_2.5_ are applied. The right column indicates mortality in 2010

Based on the PM_2.5_ regulations and proposed standards listed in Table 1, Fig. 3 summarizes the global premature mortality estimations when we apply the 8, 10, 12, 15, 20, 25, 30, 35 and 40 μg/m^3^ annual mean PM_2.5_ upper limit concentrations and the 2010 levels. This graphical representation illustrates that the reduction of mortality rates is more sensitive to lower standards (e.g., <20 μg/m^3^) compared to higher standards. The 12 μg/m^3^ limit would reduce global mortality by 15 % compared to the 15 μg/m^3^ limit, and by 27 % compared to the 20 μg/m^3^ limit, while a limit tightening from 35 to 25 μg/m^3^ would decrease global premature mortality by 10 %. We reiterate that to perform our sensitivity calculations we take into consideration that mortality caused from natural sources of PM_2.5_ cannot be controlled by air quality regulations. Our analysis shows that the relatively strong global response to PM_2.5_ reductions towards lower limits is mainly caused by the greater number of highly populated areas that would benefit from air quality control measures at these relatively low concentration levels.

Table 2 summarizes the results of our sensitivity calculations for the top 20 countries with highest PM_2.5_ mortality in 2010 and how mortality would change when applying the current EU and US air quality standards as upper limits. Table 3 presents the same information for the 28 EU member countries. Our results contribute to the body of evidence suggesting the need to adopt stricter limits for annual mean PM_2.5_ levels, like the US limit of 12 μg/m^3^ or even a lower limit to substantially reduce premature mortality in most of the world, while in strongly polluted regions like South and East Asia essentially any PM_2.5_ reduction can significantly reduce premature mortality. We reiterate that there is no strong evidence for a “safe” PM_2.5_ concentration threshold below which no health risk can be assumed (we have applied around 7.3 μg/m^3^ depending on the disease category).

## Discussion

In this work we used the integrated exposure response function (IER) of Burnett et al [23] to estimate the number of premature deaths due to PM_2.5_ air pollution induced CEV, COPD, IHD, LC (for adults ≥30 year) and ALRI (for children <5 years). The IER model is a superior predictor of RR compared to others previously used in burden assessments, to more realistically accounts for health effects at very high PM_2.5_ concentrations [23]. This is particularly relevant for regions with very high pollution levels like East and South East Asia. As we follow the method of Lelieveld et al. [8], based on Burnet et al [23] and the Global Burden of Disease – GBD 2010 [1] we also apply their uncertainty calculations and adopt their 95 % confidence interval (CI95) for PM_2.5_ related mortality. The confidence interval represents statistical uncertainty of the parameters used in the concentration response function. In previous work we derived statistical uncertainties by propagating the quantified random errors of all terms in equation 1, estimated from the 95 % confidence intervals (CI95). The uncertainties in the PM_2.5_ calculations were represented by the model simulated annual 2σ standard deviations for all model grid cells at the surface [28]. The quantified errors showed that the global mortality estimates are quite robust with an uncertainty up to about ±5 % for annual PM_2.5_ induced mortality, while at the country level the uncertainties are much larger. For uncertainty analyses and sensitivity calculations that address the shape of the health impact functions and concentration thresholds (*Xo*) we refer to analyses by Lelieveld et al. [8, 28], Burnett et al. [23] and Giannadaki et al. [19]. These issues have been also discussed by expert panels [41–44]. The existence of “safe” PM_2.5_ concentration thresholds below which no health effects occur is considered ambiguous. Scientific uncertainty about the relative toxicity of particles emitted from different source categories is one of the major weaknesses in our ability to understand the relative contributions of each source to the PM_2.5_ related mortality [45]. Studies by the Health Effect Institute suggest that certain source classes (e.g., coal combustion and traffic) should be given priority in regulation and that there is less evidence that particles from other source classes (e.g., biomass burning and natural emissions of crustal materials) increase mortality risk [46]. However, a set of usable coefficients for PM_2.5_ compounds from different sources is not available in the published literature. Lelieveld et al. [8], motivated by the reports from expert judgment studies [42–44], performed sensitivity calculations assuming that the toxicity of carbonaceous particles is five times that of inorganic and crustal compounds, maintaining the average toxicity of PM_2.5_. The expert studies indicate that aspects of the methodology and representativeness are likely to lead to several fold larger uncertainty than indicated by CI95, corroborated by the results of the sensitivity calculations on differential toxicity. While aerosol compounds such as heavy metals, soot and certain organic substances are likely to be more toxic than mineral dust and inorganic salts, they form a mixture within PM_2.5_ and cannot be treated separately based on epidemiological cohort studies. Therefore, the CI95 mentioned above for the health effects of the long-term exposure to PM_2.5_ should be considered as a lower limit of the overall uncertainty.

## Conclusions

We estimated the PM_2.5_ related premature mortality in 2010 at 3.15 million worldwide, with China ranking highest, followed by India, Pakistan, Nigeria and Bangladesh. For the EU our estimate for 2010 is 173 thousand premature deaths, and 52 thousand in the US. We performed sensitivity calculations to assess the impact of applying PM_2.5_ upper limits based on air quality standards in the EU and US, and other nationally adopted or proposed standards for annual mean PM_2.5_ pollution. Our results show that even small changes at the lower standards of annual mean PM_2.5_ concentrations could have a significant impact on mortality rates. This results from the fact that at low PM_2.5_ levels many relatively populous areas would profit from air quality improvements. Our findings underscore the large positive impact on human health by implementing the US air quality standard of 12 μg/m^3^ for annual mean PM_2.5_. Finally, we estimated the impact on mortality due to PM_2.5_ from natural sources, mainly desert dust and wild fires, which to date represents a challenge to public health in the countries in and around the dust belt. For these countries it will not be possible to meet the US and EU standards.

## Additional file

Additional file 1: Mortality calculations: Main cases and Sensitivity scenarios. (ZIP 56740 kb)

## Abbreviations

AF: Attributable fraction
ALRI: Acute lower respiratory infection
AQG: Air quality guidelines
CEV: Cerebrovascular disease
CI: Confidence interval
CIESIN: Columbia University Center for International Earth Science Information Network
COPD: Chronic obstructive pulmonary disease
ECHAM: European Centre Model Hamburg
EMAC: ECHAM/MESSy Atmospheric Chemistry, MESSy Modular Earth Submodel System
EPA: Environmental Protection Agency
GBD: Global Burden of Disease
GDP: Gross Domestic Product
IHD: Ischemic heart disease
LC: Lung cancer
PM_2.5_: Particulate Matter with an aerodynamic diameter smaller than 2.5 μm
Pop: Total population with an age of <5 years and ≥30 year
RR: Relative risk
WHO: World Health Organization
y_0_: Baseline mortality rate
ΔMort: Annual premature mortality

## References

[1] Lim SS, Vos T, Flaxman AD, Danaei G, Shibuya K (2012). A comparative risk assessment of burden of disease and injury attributable to 67 risk factors and risk factor clusters in 21 regions, 1990–2010: a systematic analysis for the Global Burden of Disease Study 2010. Lancet.

[2] Cohen AJ, Anderson HR, Ostra B, Pandey KD, Krzyzanowski M, Künzli N, Gutschmidt K, Pope A, Romieu I, Samet JM, Smith K (2005). The global burden of disease due to outdoor air pollution. J Toxicol Env Health.

[3] Ezzati M, Lopez AD, Rodgers A, Hoorn SV, Murray CJL (2002). Selected major risk factors and global and regional burden of disease. Lancet.

[4] Krewski D, Jerrett M, Burnett RT, Ma R, Hughes E, Shi Y, Turner MC, Pope III CA, Thurston G, Calle EE, Thun MJ. Extended follow-up and spatial analysis of the American Cancer Society Study linking particulate air pollution and mortality. Health Effects Institute. 2009; Boston, MA. 19627030

[5] Laden F, Schwartz J, Speizer FE, Dockery DW (2006). Reduction in fine particulate air pollution and mortality – Extended follow-up of the Harvard Six Cities Study. Am J Respir Crit Care Med.

[6] Pope CA, Ezzati M, Dockery DW (2009). Fine-particulate air pollution and life expectancy in the United States. N Engl J Med.

[7] WHO (Word Health Organization) (2009). Global health risks: mortality and burden of disease attributable to selected major risks.

[8] Lelieveld J, Evans JS, Fnais M, Giannadaki D, Pozzer A (2015). The contribution of outdoor air pollution sources to premature mortality on a global scale. Nature.

[9] WHO (World Health Organization). 7 million premature deaths annually linked to air pollution. 2014. http://www.who.int/mediacentre/news/releases/2014/air-pollution/en/. Accessed January 2016.

[10] WHO (World Health Organization). Ambient (outdoor) air quality and health. 2014. http://www.who.int/mediacentre/factsheets/fs313/en/. Accessed January 2016.

[11] WHO (World Health Organization) (2006). WHO Air Quality Guidelines for particulate matter, ozone, nitrogen dioxide and sulfur dioxide.

[12] WHO Regional Office for Europe. Health effects of particulate matter - Policy implications for countries in eastern Europe, Caucasus and central Asia. Copenhagen; 2013.

[13] Shi L, Zanobetti A, Kloog I, Coul BA, Koutrakis P, Melly SJ, Schwartz JD. Low-Concentration PM_2.5_ and Mortality: Estimating Acute and Chronic Effects in a Population-Based Study. Environ. Health. Perspect. 2015; doi:10.1289/ehp.1409111. 10.1289/ehp.1409111 PMC4710600 26038801

[14] Pinault L, Tjepkema M, Crouse D, Weichenthal S, van Donkelaar A, Martin RV, Brauer M, Chen H, Burnett RT (2016). Risk estimates of mortality attributed to low concentrations of ambient fine particulate matter in the Canadian community health survey cohort. Environ Health.

[15] EC (European Commission). Environment. 2014. http://ec.europa.eu/environment/air/index_en.htm. Accessed December 2015.

[16] WHO Regional Office for Europe, OECD. Economic cost of the health impact of air pollution in Europe: Clean air, health and wealth. Copenhagen; 2015.

[17] Bell ML, Morgenstern RD, Harrington W (2011). Quantifying the human health benefits of air pollution policies: Review of recent studies and new directions in accountability research. Environ Sci Policy.

[18] EPA (United States Environmental Protection Agency). Benefits and Costs of the Clean Air Act: 1970 to 1990. 1997. U.S. EPA.

[19] Giannadaki D, Pozzer A, Lelieveld J (2014). Modeled global effects of airborne desert dust on air quality and premature mortality. Atmos Chem Phys.

[20] Anenberg SC, Horowitz LW, Tong DQ, West JJ (2010). An estimate of the global burden of anthropogenic ozone and fine particulate matter on premature human mortality using atmospheric modeling. Environ Health Perspect.

[21] WHO (World Health Organization). World Health Organization Statistical Information System (WHOSIS), Detailed Data Files of the WHO Mortality Database. 2015. http://www.who.int/whosis/mort/download/en/index.html. Accessed May 2015.

[22] CIESIN (Columbia University Center for International Earth Science Information Network): NASA Socioeconomic Data and Applications Center (SEDAC). 2014. http://sedac.ciesin.columbia.edu/. Accessed October 2014.

[23] Burnett RT, Pope III CA, Ezzati M, Olives C, Lim SM, Mentha S, Shin HH, Singh G, Hubbell B, Brauer M, Anderson HR, Smith KR, Kan H, Laden F, Prüss-Ustün A, Turner MC, Thun M, Cohen A. An integrated risk function for estimating the Global Burden of Disease attributable to ambient fine particulate matter exposure. Environ. Health. Perspect. 2014; doi:10.1289/ehp.1307049. 10.1289/ehp.1307049 PMC3984213 24518036

[24] Pozzer A, Zimmermann P, Doering UM, van Aardenne J, Tost H, Dentener F, Janssens-Maenhout G, and Lelieveld J. Effects of business-as-usual anthropogenic emissions on air quality, Atmos. Chem. Phys. 2012b;12:6915-6937, doi:10.5194/acp-12-6915-2012

[25] Jöckel P, Tost H, Pozzer A, Brühl C, Buchholz J, Ganzeveld L, Hoor P, Kerkweg A, Lawrence MG, Sander R, Steil B, Stiller G, Tanarhte M, Taraborelli D, van Aardenne J, Lelieveld J (2006). The atmospheric chemistry general circulation model ECHAM5/MESSy: Consistent simulation of ozone from the surface to the mesosphere. Atmos Chem Phys.

[26] Pozzer A, de Meij A, Pringle KJ, Tost H, Doering UM, van Aardenne J, Lelieveld J. Distributions and regional budgets of aerosols and their precursors simulated with the EMAC chemistry-climate model, Atmos. Chem. Phys. 2012a;12:961-987, doi:10.5194/acp-12-961-2012

[27] Pozzer A, de Meij A, Yoon J, Tost H, Georgoulias AK, Astitha M (2015). AOD trends during 2001–2010 from observations and model simulations. Atmos Chem Phys.

[28] Lelieveld J, Barlas C, Giannadaki D, Pozzer A (2013). Model calculated global, regional and megacity premature mortality due to air pollution by ozone and fine particulate matter. Atmos Chem Phys.

[29] EU (European Union). Directive on ambient air quality and cleaner air for Europe (Air Quality Directive, 2008/50/EC); 2008.

[30] EPA (United States Environmental Protection Agency). Regulatory Actions. 2015. http://www.epa.gov/airquality/particlepollution/actions.html. Accessed June 2015.

[31] Canadian Ambient Air Quality Standards. 2015. http://www.ec.gc.ca/default.asp?lang=En&n=56D4043B-1&news=A4B2C28A-2DFB-4BF4-8777-ADF29B4360BD. Accessed June 2015.

[32] Australian Government, Department of the Environment and Heritage: State of the Air: Community Summary 1991-2001, ISBN 0 642 54991 5.

[33] Green J, Sánchez S (2012). Air Quality In Latin America: An Overview.

[34] CAI – Asia (Clean Air Initiative for Asia). 2015. http://cleanairinitiative.org/. Accessed June 2015.

[35] Zheng S, Pozzer A, Cao CX, Lelieveld J (2015). Long-term (2001-2012) fine particulate matter (PM_2.5_) and the impact on human health in Beijing, China, Atmos. Chem Phys.

[36] CAI – Asia (Clean Air Initiative for Asia). Particulate Matter Standards in Asia. Pasig City, Philippines; 2010.

[37] Environmental Quality Standards in Japan. 2015. http://www.env.go.jp/en/air/aq/aq.html#main. Accessed June 2015.

[38] Kingdom of Saudi Arabia: National Environmental Standard – Ambient Air Quality, Presidency of Meteorology and Environment.

[39] Krzyzanowski M, Cohen A (2008). Update of WHO air quality guidelines. Air Qual Atmos Health.

[40] Apte JS, Marshall JD, Cohen AJ, Brauer M. Addressing Global Mortality from Ambient PM_2.5_. Environ. Sci. Technol. 2015; doi:10.1021/acs.est.5b01236. 10.1021/acs.est.5b01236 26077815

[41] Cooke RM, Wilson AM, Tuomisto JT, Morales O, Tainio M, Evans JS (2007). A probabilistic characterization of the relationship between fine particulate matter and mortality: elicitation of European experts. Environ Sci Technol.

[42] Kinney PL, Roman HA, Walker KD, Richmond HM, Conner L, Hubbell BJ (2010). On the use of expert judgment to characterize uncertainties in the health benefits of regulatory controls of particulate matter. Environ Sci Policy.

[43] Roman HA, Walker KD, Walsh TL, Conner L, Richmond HM, Hubbell BJ, Kinney PL (2008). Expert judgment assessment of the mortality impact of changes in ambient fine particulate matter in the U.S. Environ Sci Technol.

[44] Tuomisto JT, Wilson A, Evans JS, Tainio M (2008). Uncertainty in mortality response to airborne fine particulate matter: Combining European air pollution experts. Rel Eng System Safety.

[45] WHO Regional Office for Europe. Review of evidence on health aspects of air pollution – REVIHAAP Project, Technical Report. Copenhagen; 2013. 27195369

[46] Lippmann M, Chen LC, Gordon T, Ito K, and Thurston GD. National Particle Component Toxicity (NPACT) initiative: Integrated epidemiologic and toxicologic studies of the health effects of particulate matter components. Health Effects Institute (HEI) Research Report 177. 2013; Boston, Massachusetts. 24377209

